# The 4^th^ National Neurology Forum | Interview with Prof. Dorel Săndesc

**DOI:** 10.25122/jml-2026-1013

**Published:** 2026-07

**Authors:** Ștefana-Andrada Dobran, Alexandra Gherman

**Affiliations:** 1 RoNeuro Institute for Neurological Research and Diagnostic, Cluj-Napoca, Romania

Interviewee: Prof. Dorel Săndesc

Interviewer: Ms. Ștefana-Andrada Dobran

Dorel Săndesc is Professor and Head of the Anesthesia and Intensive Care Department at Victor Babeș University of Medicine and Pharmacy in Timișoara, Vice-Rector of the university, and General Manager of the University County Emergency Hospital Timișoara.

He serves as President of the Expert Commission of Anesthesia and Intensive Care within the Romanian Ministry of Health and has held leadership and professional roles in the Romanian, European and international anesthesia and intensive care communities. He has contributed to the modernization of anesthesia and intensive care in Romania, including national funding programs, the adoption of European standards in education, simulation-based training, and infrastructure and equipment modernization.

He has also been involved in humanitarian and public health initiatives, including medical caravans in isolated areas and the “Vaccination Marathon” during the COVID-19 pandemic.

His scientific activity includes more than 80 articles in ISI-cited journals, seven research grants, 22 international clinical trials and three invention patents. He has received numerous distinctions, including the National Order “For Merit” and the 2022 Romanian Healthcare Awards “Doctor of the Year” Trophy.


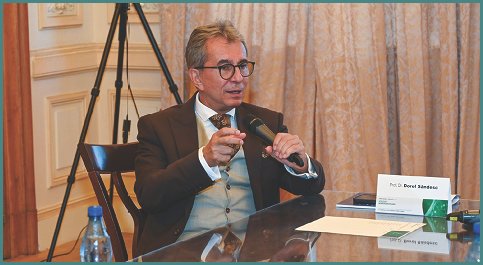



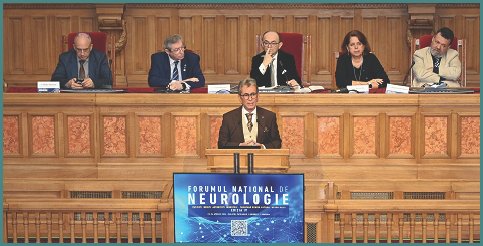


S.D.: Hello, Professor Dorel Săndesc, and welcome to the fourth edition of the National Neurology Forum in Bucharest. How do you see the role of the National Neurology Forum in fostering interdisciplinary dialogue and supporting health care reforms?

**D.S.:** It is an excellent initiative, now at the fourth edition. I think this is what we need most in modern medicine: interdisciplinary dialogue among the specialities involved in the care of neurological pathology.

Modern medicine has evolved so much that each specialty goes deeper and deeper into its own specific knowledge. Still, in doing so, it also develops a kind of specialized language that is slightly different from that of other specialties. We are, in a way, in a situation of *inter-specialty dysphasia*—we do not communicate very well.

These meetings, where different specialties come together to discuss, are therefore extremely useful. I consider this a great initiative. The fact that we bring together all the specialties involved in the care of a neurological patient is very important.

S.D.: From your perspective, what are the main challenges in the multidisciplinary management of patients with traumatic brain injury?

**D.S.:** This pathology is of utmost importance and has a huge medical, sociological, and financial impact. In [regard to the] number of deaths, the first pathologies are cardiovascular, neurovascular, and oncological.

[However], regarding the number of years of life lost, trauma–specifically neurotrauma–accounts for the highest number, because the average age of a person who died because of trauma is significantly lower than the average age of a patient who died due to chronic diseases.


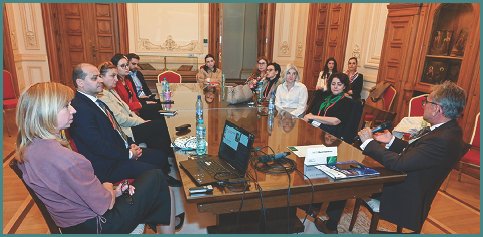



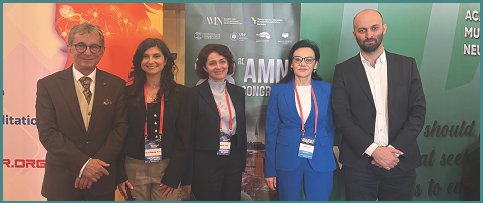


Because of this, this pathology requires special attention. Unlike cardiological, neurological, and oncological diseases, for which we already have strategies at national and international levels and specific programs, in neurotraumatology we still lack this kind of systemic approach at legal, financial, and medical levels.

This is the current situation, and, in a way, it represents a call to action–this is what is happening now in this Forum.

S.D.: How does the Academy for Multidisciplinary Neurotromatology relate to the goals that you want to achieve?

**D.S.:** The fact that this Forum is connected with the most prestigious international institutions and organizations is very important. In fact, we want to standardize care, programs, and strategies for these pathologies according to international standards.

One of the steps that we will take here is to create and consolidate the **AMN FOCUS GROUP Romania** in neurotraumatology, thereby contributing to this initiative at an international level. During the meeting, we will have three workshops on neurotraumatology with specialists from different fields. Each will come not only with the presentation of an up-to-date situation regarding medical treatment, but also with proposals for programs.

We want to build one or several or several programs for neurotraumatology and to work or fight with the system to implement it. We have experienced this, we managed to do similar programs in different fields, including my field and stage and intensive care, so I think it is achievable.


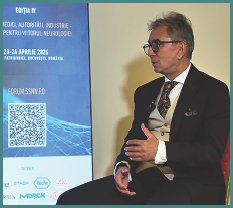


But beyond programs for the care of neurotrauma patients, we must also focus on prevention. We need proposals to improve prevention, because we have a difficult situation in Romania, with a very high number of trauma cases.

We should work on education at a general level–for example, the education for driver license to include preventive driving courses and, of course, other areas of prevention.

Prevention programs of standards of care and programs for financing and organizing the care of the patients with neurotrauma are the key priorities for advancing neurocritical care in Romania in the following years.

